# New German guidelines for the management of diverticulitis

**DOI:** 10.1002/ueg2.12331

**Published:** 2022-11-02

**Authors:** Anne F. Peery

**Affiliations:** ^1^ University of North Carolina Chapel Hill North Carolina USA

Colonic diverticulitis is a common disease and a significant burden to patients.^1^, ^2^ Acute episodes of diverticulitis are painful, debilitating, and unpredictable. Our poor understanding of diverticulitis leaves patients feeling helpless and blaming themselves for episodes. Patients fear recurrent episodes and worry they may develop a complication. Consequently, diverticulitis patients often seek advice on treatment and prevention.

Fortunately for those patients, our understanding of diverticulitis has changed dramatically in recent years. In the last decade, journals published multiple trials and observational studies on diverticulitis risk factors, disease course, and treatment. In response to this work, clinical practice is moving towards a more patient‐centered, conservative approach. In the current issue of UEG Journal, we have updated German guidelines on the management of diverticular diseases including diverticulitis.^3^, ^4^ These guidelines include several recommendations for readers to appreciate. This new work provides a timely opportunity for the practicing clinician to reconsider the treatment of diverticulitis in the context of the broader literature.

Diverticulitis management begins with an accurate diagnosis. Consistent with most existing guidelines, the German guideline authors recommend imaging be performed with ultrasound or computed tomography in patients with suspected diverticulitis (**recommendations 4.6–4.7**).^5^, ^6^, ^7^, ^8^ Experts note that imaging is most important with the first episode, with presentations concerning for a complication, and in immunocompromised patients.^9^ Because colon cancer can mimic diverticulitis, the German guideline authors recommend colonoscopy be considered based on clinical factors after the acute episode has resolved (**statement 4.12**). This is not surprising, as most guidelines now recommend colonoscopy after an episode of complicated diverticulitis to rule out malignancy.^5^, ^6^, ^7^, ^8^, ^10^ The risk of a missed cancer is higher in patients with a history of complicated diverticulitis (7.9%) compared to uncomplicated diverticulitis (1.3%).^11^

Treatment with antibiotics is no longer recommended for all patients with acute uncomplicated diverticulitis. Instead, antibiotic treatment should be selective. The German guideline authors recommend antibiotic treatment for patients with acute uncomplicated diverticulitis who are immunosuppressed, have comorbid conditions or poor overall health, or present with fever, leukocytosis, or elevated inflammatory markers (**recommendation 5.20**). Multiple practice guidelines support an antibiotic‐sparing approach to acute uncomplicated diverticulitis in patients with mild disease who are generally healthy and immunocompetent.^5^, ^6^, ^7^, ^8^, ^10^ For those who are interested, additional guidance on an antibiotic sparing approach is provided in a recent American guideline.^12^ Antibiotic treatment is mandatory for diverticulitis complicated by abscess or perforation (**recommendations 5.27 and 6.4**).

Diverging from existing guidelines, the German guideline authors suggest that mesalamine be used to treat acute uncomplicated diverticulitis (**recommendation 5.16**). No other guideline recommends mesalamine treatment for acute uncomplicated diverticulitis, and some of the risks include nausea, diarrhea, and headache. The three trials cited to support this recommendation included patients with symptomatic uncomplicated diverticular disease, not acute uncomplicated diverticulitis. Symptomatic uncomplicated diverticular disease is a debated diagnosis defined as chronic gastrointestinal symptoms in patients with diverticulosis, not diverticulitis.^13^, ^14^

Diverticulitis can become a chronic disease with unpredictable recurrences. After an episode of diverticulitis has resolved, patients look for conservative ways to reduce the risk of recurrence. The German guideline recommendations on prevention are comparable to existing guidelines. Patients with a diverticulitis should maintain a normal body weight (**recommendation 5.8**), avoid tobacco products (**recommendation 5.5**) and non‐steroidal anti‐inflammatory drugs (**recommendation 5.11**), be physically active (**recommendation 5.9**), and consume a diet high in dietary fiber (**recommendation 5.1**) and low in red meat (**recommendation 5.3**). Unfortunately, there are no medical therapies to prevent diverticulitis. Probiotics (**recommendation 5.30**), rifaximin (**recommendation 5.32**), and mesalamine (**recommendation 5.31**) are not recommended to prevent recurrence.

Elective colon resection reduces the risk of recurrent diverticulitis but comes with risks. Historically, elective resection was recommended after 1–2 episodes to prevent catastrophic recurrence. This recommendation is outdated. Guidelines now universally recommend that the number of episodes not determine whether a patient has elective resection (**recommendation 6.12**). Congruent with current guidelines, the authors recommend a patient centered approach with a focus on quality of life (**recommendation 6.13**). In contrast with the German guidelines, some authors recommend caution with elective resection to treat ongoing gastrointestinal symptoms in patients with a history of acute uncomplicated diverticulitis (**recommendation 6.2**).^15^ In a small trial, more than 60% of patients with baseline abdominal pain were still experiencing pain after elective resection for diverticulitis.^16^

As always, new guidelines are an opportunity for the practicing clinician to reconsider and advance their practice. The German authors hard work give us all the opportunity to improve the care we provide our patients with diverticulitis.

## AUTHOR CONTRIBUTIONS

Anne F. Peery concept and design, drafting, and critical revision of manuscript for content.

## CONFLICTS OF INTEREST

No relevant conflicts to report.

## Data Availability

This is an editorial.
